# The Successive Next Network as Augmented Regularization for Deformable Brain MR Image Registration

**DOI:** 10.3390/s23063208

**Published:** 2023-03-17

**Authors:** Meng Li, Shunbo Hu, Guoqiang Li, Fuchun Zhang, Jitao Li, Yue Yang, Lintao Zhang, Mingtao Liu, Yan Xu, Deqian Fu, Wenyin Zhang, Xing Wang

**Affiliations:** School of Information Science and Engineering, Linyi University, Linyi 276000, China

**Keywords:** brain image registration, generation adversarial network, deep learning

## Abstract

Deep-learning-based registration methods can not only save time but also automatically extract deep features from images. In order to obtain better registration performance, many scholars use cascade networks to realize a coarse-to-fine registration progress. However, such cascade networks will increase network parameters by an n-times multiplication factor and entail long training and testing stages. In this paper, we only use a cascade network in the training stage. Unlike others, the role of the second network is to improve the registration performance of the first network and function as an augmented regularization term in the whole process. In the training stage, the mean squared error loss function between the dense deformation field (DDF) with which the second network has been trained and the zero field is added to constrain the learned DDF such that it tends to 0 at each position and to compel the first network to conceive of a better deformation field and improve the network’s registration performance. In the testing stage, only the first network is used to estimate a better DDF; the second network is not used again. The advantages of this kind of design are reflected in two aspects: (1) it retains the good registration performance of the cascade network; (2) it retains the time efficiency of the single network in the testing stage. The experimental results show that the proposed method effectively improves the network’s registration performance compared to other state-of-the-art methods.

## 1. Introduction

Image registration is one of the basic tasks in medical image processing. It involves the acquisition of a dense deformation field (DDF) when a moving image is matched with a fixed image so that the two to-be-aligned images and their corresponding anatomical structures are aligned accurately in space [1]. The traditional registration method optimizes the cost function through a large number of iterations, a process that usually requires a significant amount of computation and time [2]. With the popularization and application of deep learning in the field of medical image registration, the deep learning registration method is now faster than the traditional image registration method. Therefore, for moving and fixed images, deformation fields can be generated by training a neural network, thus achieving rapid registration for a forward pass in the testing stage. Fan et al. [3] studied the computational costs of seven different deformable registration algorithms. The results showed that the assessed deep-learning network (BIRNet) without any iterative optimization needed the least time. Additionally, the registration accuracy improved after applying the deep learning method. For example, Cao et al. [4] proposed a deep learning method for registering brain MRI images, and it was revealed that the method’s Dice coefficient was improved in terms of registering white matter (WM), gray matter (GM), and cerebrospinal fluid (CSF).

The unsupervised learning image registration method has been widely applied because it is not difficult to obtain gold-standard registration [5]. Balakrishnan et al. [6] optimized the U-Net neural network by defining the loss function as a combination of the mean square error similarity measure and the deformation field’s smoothing constraint. de Vos et al. [7] accomplished affine and deformable registration by superimposing several networks through unsupervised training. Kim et al. [8,9] used cyclic consistency to provide implicit regularization for maintaining topology and realizing 2D or 3D image registration. Moreover, a multi-scale strategy was adopted during the experiment to solve the relevant storage problem. Jiang et al. [10] proposed an unsupervised network framework (MJ-CNN) that adopted a multi-scale joint training scheme to achieve end-to-end optimization. Kong et al. [11] designed a cascade-connected channel attention mechanism network. During cascade registration, the attention module is incorporated to learn the features of the input image, thereby improving the expression ability of the image features. Through five iterations of the deformation field, improved bidirectional image registration was realized. Yang et al. [12] used multiple cascaded U-Net models to form a network structure. In their structure, each U-Net is trained with smooth regularization parameters to improve the accuracy of 3D medical image registration. Zhu et al. [13] helped a network develop high-similarity spatial correspondence by introducing a local attention model and integrated multi-scale functionality into the attention mechanism module to achieve the coarse-to-fine registration of local information. Ouyang et al. [14] trained their designed subnetworks synergistically by training the residual recursive cascade network to realize cooperation between the subnetworks. Through the connection of the residual network, the registration speed was accelerated. Guo et al. [15] improved the image registration accuracy and efficiency of CT-MR and used two cyclic consistency methods in a full convolution neural network to generate the spatial deformation field. Sideri-Lampretsa et al. [16] considered that it was easy to obtain edge images, so they used the image’s edges to drive the multimodal registration training process and thus help the network learn more effective information. Qian et al. [17] proposed a cascade framework of a registration network, and then registered images in training stages. The authors compared the performance of the cascade network framework with the traditional registration methods, subsequently, it was determined that the registration efficiency of the proposed method was significantly improved. Golkar et al. [18] proposed a hybrid registration framework of vessel extraction and thinning for retinal image segmentation, which improved the registration accuracy of complex retinal vessels.

Inspired by the idea of two-person zero-sum game from game theory, Goodefellow et al. [19] proposed a generation adversarial network (GAN) that used two neural networks for adversarial training and continuously improved the performance of the network in all directions during a game between the two networks. In addition to the in-depth study of the generative adversarial network (GAN), the application of an adversarial network has been integrated with techniques and aims from other fields, for instance, the combination of GAN and image processing. Therefore, GANs are also widely used in image registration. Santarossa et al. [20] used generation adversarial networks combined with ranking loss for multimodal image registration. Fan et al. [21,22] implemented a GAN in the unsupervised deformable registration of 3D brain MR images. In this approach, the discrimination network identifies whether a pair of images are sufficiently similar. The resulting feedback is then used to train the registration network. Simultaneously, GANs have been applied to single- and multi-mode image registration. Zheng et al. [23] used a GAN network to realize symmetric image registration and then transformed the symmetric registration formula of single- and multi-mode images into a conditional GAN. To align a pair of single-mode images, the registration method constitutes a cyclical process of transformation from one image to another and its inverse transformation. To align images with different modes, mode conversion should be performed before registration. In the training process, the method also adopts the semi-supervised method and trains using labeled and unlabeled images. Many registration methods have been produced based on the application of generation adversarial networks [24,25,26,27,28]. Huang et al. [29] fused a difficulty perception model into a cascade neural network composed of three networks. These networks are used to predict the coarse deformation field and the fine deformation field, respectively, so as to achieve accurate registration. GANs showed excellent performance in the aforementioned studies. In the previous study, a GAN based on dual attention mechanisms was proposed, which showed good registration performance in areas with relatively flat edges, but poor registration performance in narrow and long-edge areas. To this end, based on previous research, this paper proposes a method to assist GANs in realizing the registration of long and narrow regions at the peripheries of the brain, which differs from the methods of coarse registration and fine registration. Our main contributions are summarized as follows:

1. During training, the cascade networks are trained simultaneously to save network training time.
2. The second network is used as a loss function. The mean square error loss function added to the second network can constrain the deformation field output by the second network such that it tends to 0. Only the first network is used during testing, which saves testing time.
3. Coupled with the adversarial training of GANs, the registration performance of the first network is further improved.

The rest of this paper is organized as follows. Section 2 introduces the networks proposed in this paper in detail. Section 3 introduces the experimental datasets and evaluation indicators. Section 4 introduces the experimental results obtained from the HBN and ABIDE datasets. In Section 5, we provide a discussion. Finally, the conclusions are given in Section 6.

## 2. Methodology

**Figure 1:**
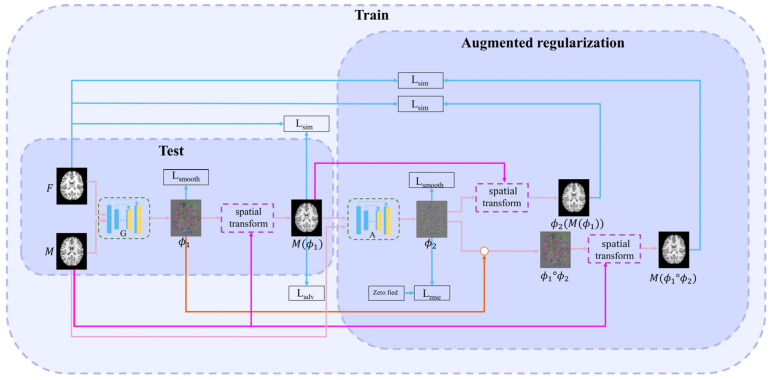
Overall network framework.

This paper proposes a method combining adversarial learning with cascade learning. Joint training of cascaded networks can allow them to predict more accurate deformation fields. The first (registration) network is used to study the deformation field $\phi_{1}$. The second (augmented) network enables the first network to learn more deformations. A discrimination network improves the first network’s performance through adversarial training. The structures of each cascading network are similar to those of VoxelMorph [6]. The proposed overall learning framework is illustrated in Figure 1.

### 2.1. First (Registration) Network

The registration network is the first network in cascading framework. Its inputs are the fixed image F and the moving image M. Its output is the deformation field $\phi_{1}$, i.e., $\phi_{1}=G(F,M)$. This network realizes the alignment from M to F, i.e., F = M($\phi_{1})$, where M($\phi_{1}$) is the warped image. Subsequently, the loss function between $M(\phi_{1})$ and F is calculated to drive the training process. This loss function includes three parts: intensity similarity loss L_sim_, adversarial loss L_adv_, and smooth regularization term L_smooth_.

The adversarial loss function of the registration network is:

(1) $$L_{\mathrm{adv}}\left(p\right)=\left\{\begin{array}{l} -\log\left(1-p\right),c\in P^{+}\\ -\log\left(p\right),c\in P^{-} \end{array}\right.$$

where p is the output value of the discrimination network and c indicates the registration network input.

Local cross-correlation metric is used to calculate the similarity of the intensity between fixed image F and warped image $M(\phi_{1})$. The specific formula of the loss function is:

(2) $$CC\left(F,M\left(\phi_{1}\right)\right)=\sum_{p\in \Omega }\frac{{\left(\sum_{p_{i}}\left(F\left(p_{i}\right)-F(p))(M\left(\phi \left(p_{i}\right)\right)-M(\phi \left(p\right))\right)\right)}^{2}}{\left({\sum_{p_{i}}\left(F\left(p_{i}\right)-F\left(p\right)\right)}^{2})({\sum_{p_{i}}\left(M\left(\phi \left(p_{i}\right)\right)-M(\phi (p))\right)}^{2}\right)}$$

where $p_{i}$ denotes the iteration of the $n^{3}$ volume center at voxel p, and Ω represents a three-dimensional voxel. In this paper, n=9 $F\left(p_{i}\right),$ and $M(\phi_{1}(p_{i}))$ represents the voxel intensities of F and $M(\phi_{1})$ at $p_{i}$, respectively. F(p) and $M(\phi_{1}(p))$ are the local mean values of $n^{3}$ volume. A higher CC indicates a more accurate alignment. According to the definition of CC, the intensity similarity loss L_sim_ is defined as follows:

(3) $$L_{\mathrm{sim}}\left(F,M\left(\phi_{1}\right)\right)=-CC(F,M(\phi_{1}))$$

Additionally, L2 regularization is implemented to smooth the deformation field $\phi_{1}$:

(4) $$L_{\mathrm{smooth}}\left(\phi_{1}\right)=\sum_{p\in \Omega }{\left\|\nabla \phi_{1}(p)\right\|}^{2}$$

### 2.2. Successive (Augmented) Network

The inputs of the successive network are F and $M(\phi_{1})$; the output is DDF $\phi_{2}$. $\phi_{2}$ is used to deform $M(\phi_{1})$ to obtain $\phi_{2}(M(\phi_{1}))$. Simultaneously, to clarify the warped image, we perform a composed operation on $\phi_{1}$ and $\phi_{2}$, i.e., $\phi_{1}{}^{\circ}\phi_{2}$. ${M(\phi }_{1}{}^{\circ}\phi_{2})$ is obtained by the moving image M with the composed DDF. Next, two intensity loss functions, namely, L_sim_($F,M{(\phi }_{1}{}^{\circ}\phi_{2})$) and L_sim_($F,\phi_{2}(M(\phi_{1}))$), are calculated between ${M(\phi }_{1}{}^{\circ}\phi_{2})$ and F and between $\phi_{2}(M(\phi_{1}))$ and F, respectively. The DDF $\phi_{2}$ is also constrained as it approaches zero deformation field through the following MSE loss function, allowing the deformation field $\phi_{1}$ to learn more accurate deformations.

The formula of MSE loss function is defined as:

(5) $$L_{\mathrm{mse}}\left(\phi_{2}\right)=\;L_{\mathrm{mse}}\left(\phi_{2},0\right)=\sum_{p\in \Omega }{\left\|\nabla \phi_{2}(p)\right\|}^{2}$$

Through this function, the output effect of the first network can achieve fine registration after the two networks are connected in series.

The loss function for the registration network is as follows:

(6) $$L_{G}=L{\mathrm{adv}}_{\;}(p)+\alpha L_{\mathrm{sim}}(F,M(\phi_{1}))+\lambda L_{\mathrm{smooth}}(\phi_{1})$$

In addition, the loss function used by the second network is:

(7) $$L_{A}=L_{\mathrm{sim}}\left(F,M\left(\phi_{1}\right)\right)+L_{\mathrm{sim}}({M(\phi }_{1}{}^{\circ}\phi_{2}))+L_{\mathrm{sim}}\left(F,\phi_{2}\left(M\left(\phi_{1}\right)\right)\right)+\;L_{\mathrm{mse}}\;\left(\phi_{2}\right)+L_{\mathrm{smooth}}\;\left(\phi_{1}\right)+L_{\mathrm{smooth}}\;\left(\phi_{2}\right)+\;L_{\mathrm{smooth}}(\phi_{1}{}^{\circ}\phi_{2})$$

The total loss function is:

(8) $$L_{\mathrm{total}}=L_{G}\;+\;L_{A}$$

### 2.3. Discrimination Network

**Figure 2:**
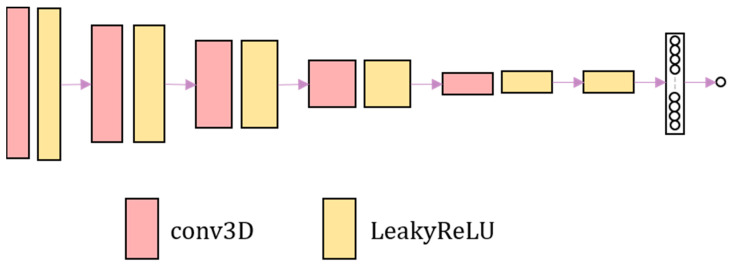
The overall framework of the adversarial network. The adversarial network consists of convolution and LeakyReLU activation layer.

The discrimination network consists of four convolutional layers combined with leakyReLU activation layers. Finally, the sigmoid activation function is used to output the probability value. The discrimination network is shown in Figure 2. The discrimination network distinguishes the authenticity of image. The harder it is to distinguish the warped image from the fixed image, the harder it is to judge the authenticity of the image by the discrimination network.

## 3. Experiment

### 3.1. Experimental Details

Python and TensorFlow were used to implement the experimental process. The program was trained and tested with GPU NVIDIA GeForce GTX 2080 Ti [30].

In the training process, the patch-based training method is adopted to reduce the occupied memory. Herein, 127 blocks are obtained from each image with a size of 182 × 218 × 182. Each block size is 64 × 64 × 64. The stride is 32. The learning rates for training the registration and discrimination networks are set to 0.00001 and 0.000001, respectively.

The traditional methods of Demons and SyN are used as comparative experiments. The deep learning model VoxelMorph is also trained. VoxelMorph is a model of medical image registration based on unsupervised learning. Therefore, VoxelMorph is selected as the comparative experiment for deep learning. The Dice score, structural similarity, and Pearson’s correlation coefficient are used as the evaluation indicators to verify the superiority of the experimental results. Moreover, the influence of the MSE and L_sim_ loss functions on the experimental results is investigated.

### 3.2. Datasets

To prove the flexibility and superior performance of the proposed method, the HBN [31] and ABIDE datasets [32] are used for training and testing. The HBN dataset consists of brain data obtained from patients with ADHD (aged 5–21 years). Herein, 496 and 31 T1-weighted brain images are selected for training and testing, respectively. ABIDE is a dataset consisting of brain images from patients with autism (aged 5–64 years). Herein, 928 and 60 T1-weighted brain images are used for training and testing, respectively. The fixed image used in training comprises a pair of images randomly selected from the training set such that each image is linearly aligned to the fixed image. The image size of both the HBN and ABIDE datasets is 182 × 218 × 182 voxels with a resolution of 1 × 1 × 1 mm^3^. Both these datasets contain segmentation marker images of CSF, GM, and WM.

### 3.3. Evaluation Indicators

#### 3.3.1. Dice Score

The Dice coefficient (Dice) index is used to evaluate the degree of overlap between a warped segmentation image and the segmentation image of the fixed image. This index reflects the similarity between the experimental and the standard segmentation images. It is defined as follows:

(9) $$Dice=2\left|\frac{X_{seg}\cap Y_{seg}}{X_{seg}\cup Y_{seg}}\right|$$

where $X_{seg}$ and $Y_{seg}$ represent the standard and warped segmentation images, respectively. The range of Dice values is 0–1, corresponding to a range in the gap between the warped and the standard segmentation images progressing from large to small values, respectively. Alternatively, the closer the experimental result is to 1, the more similar the warped segmentation image is to the standard segmentation image, and the better is the registration result.

#### 3.3.2. Structural Similarity

The structure similarity index measure [33] can measure the similarity of two images. The SSIM is calculated as:

(10) $$\mathrm{SSIM}\left(X,Y\right)=\frac{\left(2\mu_{X}\mu_{Y}+c_{1})(2\sigma_{XY}+c_{2}\right)}{\left(\mu_{X}^{2}+\mu_{Y}^{2}+c_{1})(\sigma_{X}^{2}+\sigma_{Y}^{2}+c_{2}\right)}$$

where X, Y represent the two input 3D images; $\mu_{X}$ and $\mu_{Y}$ represent the average value of X and Y, respectively. $\sigma_{X}^{2}$ and $\sigma_{Y}^{2}$ are the variances of X and Y, respectively. $\sigma_{X}$ and $\sigma_{Y}$ represent the standard deviation of X and Y, respectively. $\sigma_{XY}$ represents the covariance of X and Y. $c_{1}$ and $c_{2}$ are constants used to avoid system errors caused by a denominator equal to 0. The SSIM can measure the structural similarity between the real and warped images. A SSIM value close to 1 indicates that the two images have a high degree of similarity.

#### 3.3.3. Pearson’s Correlation Coefficient

Pearson’s correlation coefficient (PCC) was used to measure the similarity between two 3D images. The calculation formula of PCC is:

(11) $$\rho (X,Y)\frac{\sum_{i=1}^{n}\left(X_{i}-\overset{-}{X})(Y_{i}-\overset{-}{Y}\right)}{\sqrt{\sum_{i=1}^{n}{\left(X_{i}-\overset{-}{X}\right)}^{2}}\sqrt{\sum_{i=1}^{n}{\left(Y_{i}-\overset{-}{Y}\right)}^{2}}}$$

The closer the value of PCC is to 1, the greater is the correlation. A PCC of 0 indicates no correlation. X, Y refer to the two input 3D images. $\overset{-}{X}$ and $\overset{-}{Y}$ represent the mean value of X and Y, respectively.

## 4. Results

The proposed methodology is compared with the following approaches: (1) Demons and SyN, two traditional registration methods; (2) Voxelmorph (VM), an unsupervised deep learning registration method; and (3) VM + A, a method consisting of a simultaneously trained registration network and augmented network.

**Figure 3:**
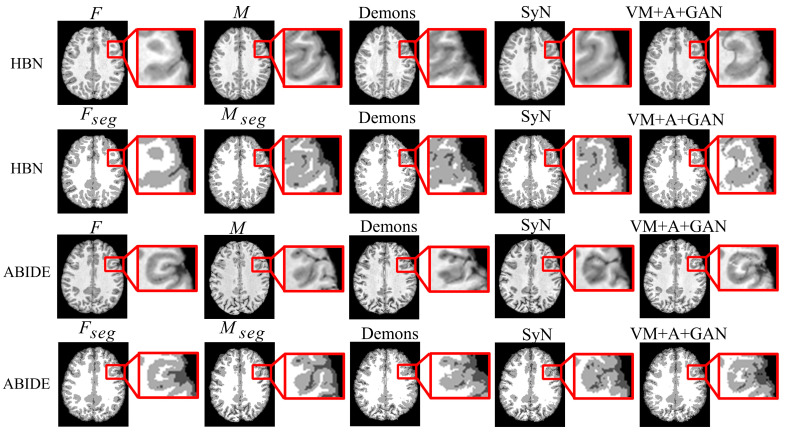
Registration results of Demons, SyN, and our proposed method using HBN and ABIDE datasets.

First, the proposed GAN method (VM + A + GAN) is compared with Demons and SyN, which are two traditional methods. Table 1 and Table 2 summarize the test results obtained through different datasets, and all indicators show that our experimental results are the best. Figure 3 shows the comparison of the test results of the two datasets. The first row of the experimental image represents the original image obtained from the HBN dataset, and the second row represents the segmentation image corresponding to the original image derived from the HBN dataset. Similarly, the third row represents the original image based on the ABIDE dataset, and the fourth row represents the segmentation image corresponding to the original image derived from the ABIDE dataset. Compared with Demons and SyN, the image obtained by the proposed GAN method is closer in appearance to the fixed image, and the parts with differences are shown in the enlarged image on the right.

**Figure 4:**
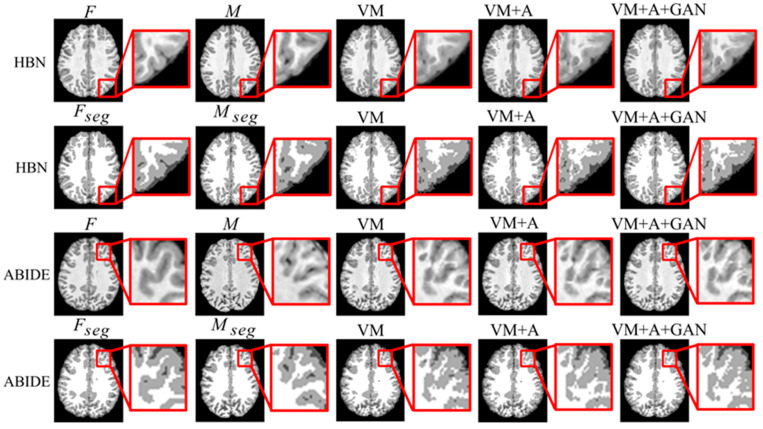
Registration results based on deep learning methods. Among them, VM represents the result obtained by the VoxelMorph method, VM + A represents the result obtained by training the registration network and the enhanced network together, and VM + A + GAN represents the result obtained by our method.

Second, the proposed GAN method is compared with the VM and VM + A methods. Figure 4 shows the registered moving image and the fixed image. Moreover, the first row represents the original image from the HBN dataset, and the second row represents the segmentation image corresponding to the original image from the HBN dataset. Similarly, the third row represents the original image from the ABIDE dataset, and the fourth row represents the segmentation image corresponding to the original image from the ABIDE dataset. Additionally, the enlarged figure on the right shows that the result for the proposed method regarding the training of the registration, augmented, and discrimination networks together is closer to the fixed image. Through the experimental results, the performance of the registration, augmented, and discrimination networks when trained together is verifiably better than that of the registration network trained individually and of the registration and augmented networks trained simultaneously.

**Figure 5:**
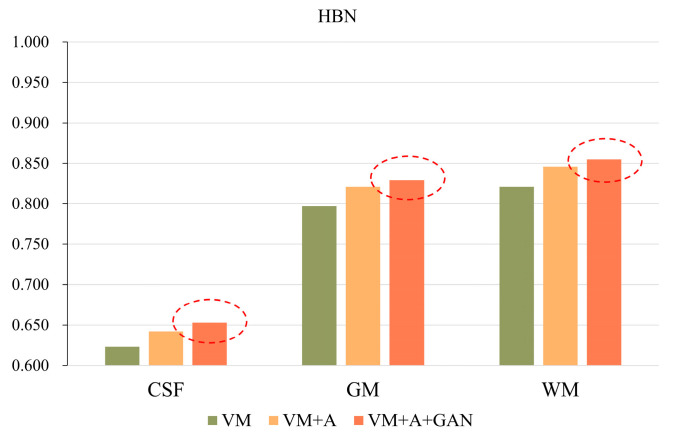
GAN registration performance on the HBN dataset.

**Figure 6:**
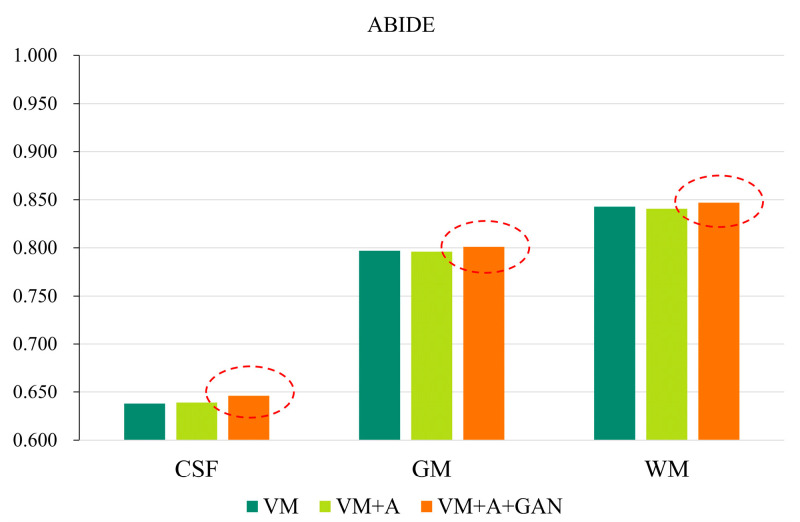
GAN registration performance on the ABIDE dataset.

In order to more clearly highlight the effectiveness of the method proposed in this paper, Figure 5 shows the experimental results of the three parts of the brain tissue based on the HBN dataset, and Figure 6 shows the experimental results of the three parts of the brain tissue based on the ABIDE dataset. The dotted circle in the figure is the result obtained by the method proposed in this paper.

Table 3 and Table 4 summarize the Dice, SSIM, and PCC indices corresponding to the different datasets. Considering Table 3, for the HBN dataset, the proposed method improves the precision values by 0.030, 0.032, and 0.034 compared with the VM method. For the ABIDE dataset, the proposed method improves the accuracies by 0.008, 0.004, and 0.004 compared with the VM method. Considering Table 4, for the HBN dataset, the proposed method increases the SSIM and PCC indices by 0.02 and 0.008, respectively, compared with the VM method. For the ABIDE dataset, the proposed method improves the SSIM and PCC indices by 0.006 and 0.003, respectively, compared with the VM method.

## 5. Discussion

The usage of a registration and discrimination networks for image registration is a common method. Such a registration method has been investigated experimentally in previous work [34]. However, this adversarial method for training a GAN only limitedly improves a registration network’s performance, and the registration capacity in some narrow and long edge areas needs to be further improved. Therefore, this paper proposes a method of training three networks together to allow the registration network to learn more deformations, further improving the registration performance. When the three networks are trained together, the use of different loss functions has a certain impact on the experimental results, which is discussed in the following subsections.

### 5.1. Importance of MSE

**Figure 7:**
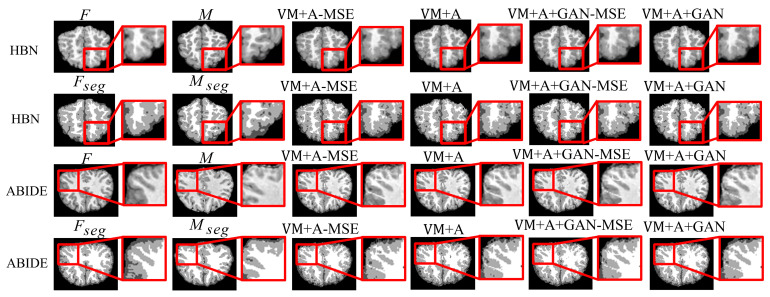
Experimental results regarding the use of the MSE loss function when employing the HBN and ABIDE datasets. Among them, VM + A − MSE indicates that the MSE loss function has been removed when training the registration network and the enhanced network, VM + A indicates the experimental results when the MSE loss function is retained when training the registration network and the enhanced network, VM + A + GAN − MSE indicates our method’s experimental results following the removal of the MSE loss function, and VM + A + GAN represents our experimental results with the MSE loss function retained.

When two networks (VM + A) were trained together, both the L_smooth_ loss function of the deformation field $\phi_{2}$ and the MSE loss function were calculated. An experiment was also performed without the MSE loss function (VM + A − MSE) to verify its effectiveness. Additionally, when the three networks (VM + A + GAN) were trained together, the MSE loss function was removed again (VM + A + GAN − MSE), and experiments were performed to verify the impact of the MSE loss function on the experimental results. Through comparison, the best registration effect was achieved when the three networks were trained together and combined with the MSE loss function. The results are shown in Figure 7.

Table 5 summarizes the experimental results regarding the removal of the MSE loss function (VM + A − MSE) when two networks were trained together (VM + A) and the removal of the MSE loss function (VM + A + GAN − MSE) when three networks were trained together (VM + A + GAN). When comparing the results, note that the removal of the MSE loss function reduces registration accuracy, thus verifying that registration performance can be improved by adding the MSE loss function when these three networks are trained together. Comparing the SSIM and PCC metrics in Table 6, the loss function used by the proposed method achieves good results. Figure 4 shows the comparison of the experimental results after the MSE loss function was removed (VM + A − MSE) when two networks were trained together and after the MSE loss function was removed (VM + A + GAN − MSE) when three networks were trained together. Evidently, the proposed method obtained a result that is closer to the fixed image, which confirms the effectiveness of training three networks simultaneously; moreover, note that the proposed method intuitively shows a good registration effect in the narrow and long regions of the peripheries of the brain images. The first row of the resulting images represents the original image from the experimental results for the HBN dataset, and the second row represents the segmentation image corresponding to the original image from the experimental results for the HBN dataset. Similarly, the third row represents the original image from the experimental results for the ABIDE dataset, and the second row represents the segmentation image corresponding to the original image from the experimental results for the ABIDE dataset.

### 5.2. Importance of L_sim_

**Figure 8:**
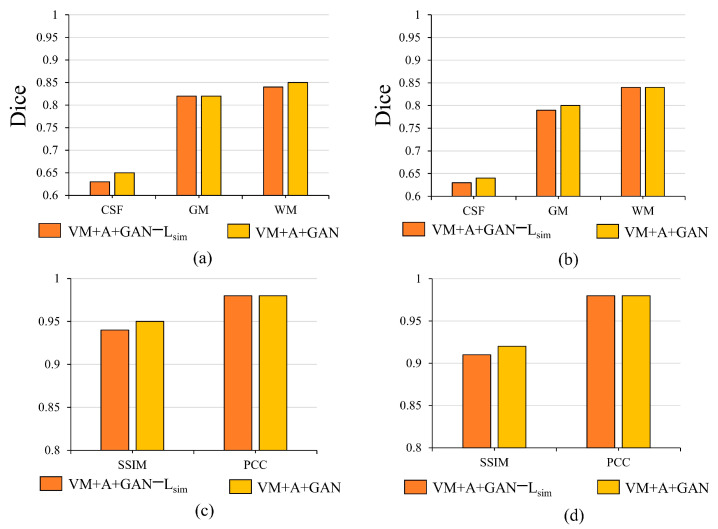
Influence of L_sim_ loss function on registration results.

When the three networks (VM + A + GAN) are trained together, the L_smooth_ loss functions between the $\phi_{2}(M(\phi_{1}))$ image and the fixed image F as well as the $M(\phi_{1}{}^{\circ}\phi_{2})$ image and the fixed image F are removed for experimental comparison. After removing the two L_sim_ loss functions, the registration accuracy decreases significantly. Through this experimental analysis, it is evident that the L_sim_ loss function can restrict the similarity among the images to a certain extent, which proves the effectiveness of adding the L_sim_ loss function. By observing the histogram in Figure 8, it is evident that the proposed method improves the Dice, SSIM, and PCC indices. In Figure 8, note that (a) shows the importance of verifying the L_sim_ loss function for the HBN dataset; (b) shows the difference between verifying the proposed method for the ABIDE dataset and removing the L_sim_ loss function in the Dice index; (c) shows the impact of removing the L_sim_ loss function on the SSIM and PCC indices for the HBN dataset; and (d) shows the impact of removing the L_sim_ loss function on the SSIM and PCC indices for the ABIDE dataset.

### 5.3. Importance of Different Deformation Fields

The Dice values for when two networks were trained simultaneously are calculated and discussed next to verify $\phi_{1}$, $\phi_{2}$, and $\phi_{1}{}^{\circ}\phi_{2}$ in the images.

For $\phi_{1}$, the similarity is calculated between the warped moving image segmentation image $M_{seg}(\phi_{1})$ and the fixed image segmentation image $F_{seg}$, expressed as $M_{seg}\left(\phi_{1}\right)-F_{seg}$. For $\phi_{2}$, the similarity is calculated between the warped $\left(M_{seg}\left(\phi_{1}\right))(\phi_{2}\right)$ and the fixed image segmentation image $F_{seg}$, expressed as $\left(M_{seg}\left(\phi_{1}\right)\right)\left(\phi_{2}\right)-F_{seg}$. For $\phi_{1}{}^{\circ}\phi_{2}$, the similarity is calculated between the warped moving image segmentation image $M_{seg}\left(\phi_{1}{}^{\circ}\phi_{2}\right)$ and the fixed image segmentation image $F_{seg}$, expressed as $M_{seg}\left(\phi_{1}{}^{\circ}\phi_{2}\right)-F_{seg}$.

Considering the Dice values in the Table 7, the deformation field ($\phi_{2})$ still plays a certain role in image registration, but a significantly miniscule role. Therefore, the registration network still allows the deformation field ($\phi_{1}$) to learn more deformations, and the augmented network only plays a secondary role.

## 6. Conclusions

In this paper, a method wherein three networks (registration, augmented, and discrimination networks) are trained together is proposed, for which the MSE loss function is introduced into the augmented network to improve the registration network’s performance. It was demonstrated that the registration network’s performance was further improved when coupled with the adversarial capacity of a GAN. Then, it was proven that the proposed method offers significant advantages over the existing methods. In addition, it was clarified that the proposed training method is easy to implement, and that the implemented loss function is easy to obtain.

In the future, a more novel GAN will be used to further improve image registration performance; moreover, more indicators will be used for comparison. The developed model will then be tested on different datasets to prove its excellent generalizability.

## Figures and Tables

**Table 1:** Dice values obtained with the HBN and ABIDE datasets. Bold numbers indicate the best results.

	HBN			ABIDE		
**Methods**	**CSF**	**GM**	**WM**	**CSF**	**GM**	**WM**
Demons	0.513 ± 0.158	0.335 ± 0.320	0.345 ± 0.330	0.410 ± 0.228	0.312 ± 0.305	0.332 ± 0.320
SyN	0.585 ± 0.026	0.768 ± 0.022	0.786 ± 0.015	0.593 ± 0.041	0.749 ± 0.019	0.791 ± 0.023
VM + A + GAN	**0.653 ± 0.041**	**0.829 ± 0.029**	**0.855 ± 0.015**	**0.646 ± 0.046**	**0.801 ± 0.024**	**0.847 ± 0.019**

**Table 2:** SSIM and PCC metrics obtained with the HBN and ABIDE datasets. Bold numbers indicate the best results.

	HBN		ABIDE	
**Methods**	**SSIM**	**PCC**	**SSIM**	**PCC**
Demons	0.781	0.886	0.763	0.886
SyN	0.904	0.962	0.870	0.958
VM + A + GAN	**0.956**	**0.984**	**0.920**	**0.985**

**Table 3:** Dice indicator based on deep learning. Bold numbers indicate the best results.

	HBN			ABIDE		
**Methods**	**CSF**	**GM**	**WM**	**CSF**	**GM**	**WM**
VM	0.623 ± 0.037	0.797 ± 0.027	0.821 ± 0.015	0.638 ± 0.048	0.797 ± 0.024	0.843 ± 0.018
VM + A	0.642 ± 0.042	0.821 ± 0.029	0.846 ± 0.014	0.639 ± 0.048	0.796 ± 0.023	0.841 ± 0.018
VM + A + GAN	**0.653 ± 0.041**	**0.829 ± 0.029**	**0.855 ± 0.015**	**0.646 ± 0.046**	**0.801 ± 0.024**	**0.847 ± 0.019**

**Table 4:** SSIM and PCC metrics for deep-learning-based registration methods. Bold numbers indicate the best results.

	HBN		ABIDE	
**Methods**	**SSIM**	**PCC**	**SSIM**	**PCC**
VM	0.936	0.976	0.914	0.982
VM + A	0.936	0.976	0.914	0.982
VM + A + GAN	**0.956**	**0.984**	**0.920**	**0.985**

**Table 5:** Dice values when using the MSE loss function and the HBN and ABIDE datasets. Bold numbers indicate the best results.

Methods	HBN			ABIDE		
	CSF	GM	WM	CSF	GM	WM
VM + A − MSE	0.641 ± 0.041	0.821 ± 0.029	0.840 ± 0.014	0.638 ± 0.048	0.797 ± 0.024	0.843 ± 0.018
VM + A	0.642 ± 0.042	0.821 ± 0.029	0.846 ± 0.014	0.639 ± 0.048	0.796 ± 0.023	0.841 ± 0.018
VM + A + GAN − MSE	0.652 ± 0.041	0.829 ± 0.028	0.854 ± 0.014	0.645 ± 0.047	0.800 ± 0.024	0.846 ± 0.019
VM + A + GAN	**0.653 ± 0.041**	**0.829 ± 0.029**	**0.855 ± 0.015**	**0.646 ± 0.046**	**0.801 ± 0.024**	**0.847 ± 0.019**

**Table 6:** SSIM and PCC values when using the MSE loss function and the HBN and ABIDE datasets. Bold numbers indicate the best results.

Methods	HBN		ABIDE	
	SSIM	PCC	SSIM	PCC
VM + A − MSE	0.950	0.982	0.911	0.982
VM + A	0.952	0.983	0.910	0.981
VM + A + GAN − MSE	**0.957**	**0.985**	0.919	0.985
VM + A + GAN	0.956	0.984	**0.920**	**0.985**

**Table 7:** Test results of output images from registration and augmented network for two datasets. Bold numbers indicate the best results.

Methods	HBN			ABIDE		
	CSF	GM	WM	CSF	GM	WM
$M_{seg}\left(\phi_{1}\right)-F_{seg}$	0.642 ± 0.042	0.821 ± 0.029	0.846 ± 0.014	0.639 ± 0.048	0.796 ± 0.023	0.841 ± 0.018
$\left(M_{seg}\left(\phi_{1}\right)\right)\left(\phi_{2}\right)-F_{seg}$	**0.644 ± 0.042**	**0.825 ± 0.030**	**0.853 ± 0.015**	**0.642 ± 0.048**	**0.802 ± 0.023**	**0.848 ± 0.018**
$M_{seg}\left(\phi_{1}{}^{\circ}\phi_{2}\right)-F_{seg}$	0.643 ± 0.042	0.821 ± 0.028	0.845 ± 0.013	0.640 ± 0.048	0.796 ± 0.023	0.839 ± 0.018

## Data Availability

All the datasets used to train the model presented in this paper were obtained from the Internet.
